# Comparison of Hybrid Machine Learning Approaches for Surrogate Modeling Part Shrinkage in Injection Molding

**DOI:** 10.3390/polym16172465

**Published:** 2024-08-29

**Authors:** Manuel Wenzel, Sven Robert Raisch, Mauritius Schmitz, Christian Hopmann

**Affiliations:** 1 Corporate Research, Robert Bosch GmbH, Robert-Bosch-Campus 1, 71272 Renningen, Germany; 2 Institute for Plastics Processing (IKV) in Industry and Craft at RWTH Aachen University, Seffenter Weg 201, 52074 Aachen, Germany; mauritius.schmitz@ikv.rwth-aachen.de (M.S.); christian.hopmann@ikv.rwth-aachen.de (C.H.)

**Keywords:** hybrid machine learning, hybrid modeling patterns, injection molding, surrogate model, shrinkage

## Abstract

Machine learning (ML) methods present a valuable opportunity for modeling the non-linear behavior of the injection molding process. They have the potential to predict how various process and material parameters affect the quality of the resulting parts. However, the dynamic nature of the injection molding process and the challenges associated with collecting process data remain significant obstacles for the application of ML methods. To address this, within this study, hybrid approaches are compared that combine process data with additional process knowledge, such as constitutive equations and high-fidelity numerical simulations. The hybrid modeling approaches include feature learning, fine-tuning, delta-modeling, preprocessing, and using physical constraints, as well as combinations of the individual approaches. To train and validate the hybrid models, both the experimental and simulated shrinkage data of an injection-molded part are utilized. While all hybrid approaches outperform the purely data-based model, the fine-tuning approach yields the best result in the simulation setting. The combination of calibrating a physical model (feature learning) and incorporating it implicitly into the training process (physical constraints) outperforms the other approaches in the experimental setting.

## 1. Introduction

Injection molding is a processing technique widely utilized to produce plastic components. Its ability to achieve short cycle times and manufacture complex geometries has made it a preferred choice for high-volume production in various industries [1]. However, as customer demands continue to rise, manufacturers face the challenge of maintaining and improving the quality of injection-molded parts. This necessitates the optimization and monitoring of the injection molding process, which can be achieved through the application of ML methods and modeling techniques.

ML methods offer immense potential in optimizing the injection molding process by uncovering the underlying relationships between process feedback (e.g., cavity sensors) or process settings (e.g., set holding pressure) and the resulting quality attributes (e.g., dimensions). By learning these relationships, ML models can predict the resulting part quality and, subsequently, optimize or control the quality by determining the optimal process settings [2]. Examples of surrogate models in injection modeling include the optimization of mechanical properties [3], shrinkage and warpage [4,5], or in model predictive control [6,7].

Neural networks (NN) have gained significant popularity in recent years, particularly in areas such as vision [8] and speech applications [9]. In these domains, supervised training procedures are commonly employed, minimizing the discrepancy between the network’s predictions and the training data. However, in scientific and engineering fields, generating the necessary amount of training data can be a complex, time-consuming, and costly endeavor, especially when dealing with complex nonlinear relationships and incorporating real-world trials.

In the context of thermoplastic injection molding, Design of Experiments (DoE) is frequently employed to generate the required data for modeling the relationships between process and machine parameters and quality attributes. However, the learned relationships of data-driven models are statistical in nature and lack physical insights. Additionally, classical data-driven ML methods lack robustness or fail to generalize when confronted with partial information i.e., small datasets or when trying to extrapolate [10]. A potential solution is the combination of ML methods with physics-based domain knowledge, also known as hybrid modeling [11].

This study aims to reduce the amount of data needed to establish a surrogate model for shirnkage prediction in the injection molding process by improving their generalizability. Since the optimization of the shrinkage and warpage of injection-molded parts is a commonly performed task [5], the focus of the study is modeling the resulting part width depending on the process settings. To achieve improved generalization, various hybrid modeling patterns are evaluated by combining the underlying physics, via a process simulation, and data into the training of a surrogate model.

This paper is structured in the following way. First, a brief background in shrinkage prediction for polymers in injection molding is provided, as well as an introduction into hybrid modeling. Next, the used specimen and the setup of the hybrid model is presented together with the simulation and experimental data used for calibrating and validating the approach. Lastly, the results of the hybrid approaches are investigated and possible future extensions are discussed.

## 2. State of the Art

### 2.1. Physics-Based Shrinkage Prediction

**Figure 1:**
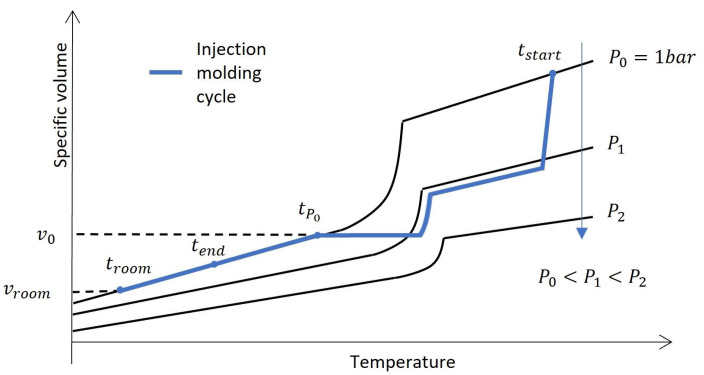
Example of an injection molding cycle within a PVT diagram for semi-crystalline thermoplastics.

For the prediction of the resulting part dimensions in injection molding, two approaches are commonly utilized: PVT models and residual stress or strain models [12]. PVT models estimate the free volumetric shrinkage of the part after it detaches from the cavity wall. A typical injection molding cycle is depicted on a PVT plot in Figure 1. The shrinkage is estimated using the relative reduction in the specific volume between the two time points $t_{P_{0}}$ and $t_{room}$, where $t_{P_{0}}$ is the point where the pressure reaches ambient pressure (P=1 bar).

To account for the effects of shrinkage and warpage, thermoelastic stress–strain models can be employed for the displacement calculation, given by Equation (4) [12]:

(1) $$\sigma_{ij}=C_{ijkl}\left(\epsilon_{kl}^{\mathrm{total}}-\epsilon_{kl}^{\mathrm{th}}\right)+\sigma_{I_{ij}}$$

The displacement field *u* is determined by solving the following Equation (2), subject to appropriate boundary conditions [12]:

(2) ∇·σ=0

For instance, in the case of a detached part, the free boundary condition applies as follows [12]:

(3) $$\sigma_{ij}n=0$$

Within these equations, $\epsilon_{kl}^{\mathrm{total}}$ represents the total elastic strain, while $\epsilon_{kl}^{\mathrm{th}}$ denotes the thermal strain from free quench during cooling after the part is ejected. The term $\sigma_{ij}$ represents the total stress and $\sigma_{I_{ij}}$ represents the initial stress, i.e., thermally and pressure-induced residual stresses generated during cooling inside the mold. Lastly, $C_{ijkl}$ is the strain–stress matrix, coupling the two. The constitutive strain–displacement relationship is expressed as follows:

(4) $$\epsilon_{xx}\left(x,\theta \right)=\frac{du_{x}}{dx}$$

Commercial simulation software is typically used to create physics-based shrinkage and warpage predictions. For instance, ref. [13] conducted a study to identify the design variables that have a significant impact on warpage and volume shrinkage in the injection molding process. They employed the response surface method (RSM), MoldFlow^®^ Insight^®^ 2004.5 simulation, and statistical analysis of variance (ANOVA) to analyze the effects of various parameters. A central composite design with 30 runs was used to create the response surface. Their results indicated that the melt temperature had the highest influence on dimension shrinkage in the transverse direction, followed by packing pressure, mold temperature, and injection velocity. Similarly, ref. [4] conducted a separate study to identify the design variables that significantly impact warpage and volumetric shrinkage in the injection molding process. They also used Moldflow^®^ simulations, RSM, and ANOVA to analyze the effects of various parameters. They used an orthogonal design with six factors and five levels, with a total of sixty-nine samples. Their findings revealed that the melt temperature, holding time, injection time, and cooling time were the most influential factors affecting the outcomes of the study.

While modern simulation tools are capable of predicting the primary effects of process settings on shrinkage and warpage, they often neglect microscale effects, leading to discrepancies between the simulated and experimental results. While the thermal strain due to free quench during cooling can be accurately estimated with the Coefficient of Thermal Expansion (CTE), the estimation of the residual stresses $\sigma_{I_{ij}}$ formed within the processes poses a challenge. To tackle this issue, some commercial simulation softwares have adopted their own strategies. Among others, Autodesk Moldflow Insight 2021.1^®^ uses a PVT-scaled approach by default for the displacement calculation, which is proprietary knowledge, and the exact calculation is not known to the public. Another example is the developed Corrected In-Mold Residual Stress (CRIMS) model, which gives a better estimation of the residual stresses developed during the molding process [14]. Furthermore, efforts have been made to develop more accurate material models, including improved PVT models and crystallization models. For instance, ref. [15] incorporated microscale properties into standard PVT models, utilizing the Two-Domain Tait Equation, to address this issue, resulting in more accurate simulations. However, it is important to note that these approaches require time and effort to calibrate and implement.

### 2.2. Data-Driven Surrogate Models

The creation of data-driven surrogate models for the injection molding process with NNs dates back to the 90s, where networks were first trained to model the relationships between process parameters and final quality attributes. The use of NNs enables the establishment of an approximate function to estimate the non-linear relationships between design variables and quality indicators. Subsequently, numerous studies have been published, wherein networks have been trained to learn the relationship between process inputs and final dimensions. These surrogate models play a pivotal role in the workflow of injection molding optimization, offering a computationally efficient method to explore the input design space. Furthermore, the precision of the subsequent optimization is directly influenced by the accuracy of these predictive models.

For example, in the study by [4], in addition to RSM, NNs with two hidden layers were employed to construct prediction models capable of handling the non-linear relationship between input variables and shrinkage and warpage. Similarly, in [16,17], a Taguchi experimental design and ANOVA method were initially used to investigate the impact of process settings on shrinkage, followed by training a simple NN to create a surrogate model which can then be used for optimization routines. Furthermore, ref. [18] compared different DoEs for generating datasets for a NN and polynomial regression models, finding that a $2^{6-3}$ fractional factorial design with a center point was the most efficient, while a central composite design was the most effective. However, data acquisition poses a challenge in injection molding due to the wide range of processes and phenomena involved. Extensive DoEs can be costly, and, in regular production, only limited variation is expected. Additionally, in the early development phases, only small datasets are typically available. Regardless of the input variables, the relationships established between input and output are purely data-driven and lack a comprehensive physical understanding of the injection molding process.

### 2.3. Hybrid Modeling Patterns

In recent years, the integration of domain knowledge into ML algorithms [11], also known as hybrid modeling, has gained significant attention to address the need for large datasets for initial training and model updating. A recent article by [19] has provided a comprehensive summary and formalization of frequently used hybrid models, deriving reusable patterns from them. The term “patterns” was chosen by the authors to denote the different types of base patterns that can be combined to create more complex hybrid models. In their paper, they presented four patterns as follows: physics-based preprocessing (PP), delta model (DM), feature learning (FL), and physical constraints (PC). Additionally, this work explores a fine-tuning (FT) approach. The following short descriptions and mathematical formalizations of combining data-driven models D(θ) with physics-based models P(θ) to create hybrid models H(θ) is based on the work of [19] and has been extended to incorporate the FT approach.

#### 2.3.1. Physics-Based Preprocessing (PP)

Physics-based preprocessing steps are commonly employed in practice, such as in physics-based feature engineering. The inputs θ undergo a series of physics-inspired transformations, which are then additionally fed into the data-based model. Mathematically, P(θ) is utilized as an additional input as follows:

(5) H(θ)=D(θ,P(θ))

This patternis applied in injection molding, for instance for dimensionality reductions in sensor data. Physically interpretable pressure integrals over the injection and holding phase are utilized for this purpose, owing to their high correlation with weight and dimensional features (see e.g., [20]).

#### 2.3.2. Delta Model (DM)

In scenarios where an initial prediction can be made based on a physical model, the data-based model can be employed to learn the error between the physics-based prediction and the observations. The final prediction can be obtained by combining the two models:

(6) H(θ)=D(θ)+P(θ)

Despite the easy-to-implement approach, the authors are not aware of any publications that have applied this method specifically for quality prediction in injection molding.

#### 2.3.3. Feature Learning (FL)

Instead of utilizing data solely to assess the error of the physics-based model, as in the delta model, an alternative approach involves using data to calibrate the physical model. In this context, the “D” index signifies the calibration, with the prediction still being made by a physics-based model:

(7) $$H\left(\theta \right)=P_{D}\left(\theta \right)$$

For instance, in the field of injection molding, ref. [21] employed pressure data from an actual process to calibrate material coefficients for simulation by identifying matching simulated and real pressure curves.

#### 2.3.4. Physical Constraints (PC)

Physical constraints are used to inform the architecture or learning process of a data-driven model. The constraints can affect the structure of the model, its parameters, or its computational results. The hybrid model is formed by incorporating these constraints either directly on the final outputs or intermediate results. The general form is denoted by the following equation:

(8) $$H\left(\theta \right)=D_{P}\left(\theta \right)$$

Furthermore, a distinction is made between hard and soft constraints. Hard constraints are implemented to ensure that the hybrid model cannot violate the constraints, while soft constraints are typically expressed as physics-informed losses that guide predictions to fall within a desired range.

An example of a hard constraint is the use of a softmax activation function, where the final prediction cannot violate the desired constraint, such as becoming negative [19]. Implicit knowledge utilization has been explored in the work of [22], where conditional physics-informed neural networks (PINNs) were employed to develop a surrogate model of the part temperature during the cooling within the injection molding process. This study demonstrated that the effects of process parameters could be implicitly learned using only physics-informed loss functions.

#### 2.3.5. Fine-Tuning (FT)

The fine-tuning approach is a key hybrid modeling pattern that involves the pre-training of the network on data with similar physics, enabling a subsequent fine-tuning or transfer-learning [23] step with fewer data samples. This process can be represented as follows:

(9) w=D(θ)←P(θ)

An illustrative example of this hybrid modeling pattern in the context of injection molding is the application of a transfer-learning approach to share information between simulation and real processes [24] or different materials [25] using pretrained models.

### 2.4. Summary

The field of shrinkage prediction in injection molding has seen significant advancements, but there remains a need for further research to enhance the accuracy and efficiency of current approaches. While physics-based methods have made progress, they still struggle to capture microscale effects and fully account for the complex material behavior in the process. Data-driven methods, such as NNs, have been widely used but are limited by the availability of data and the lack of physical understanding. Consequently, there is a need to implement hybrid approaches for shrinkage prediction that integrate the knowledge of the underlying physics. Although individual hybrid modeling approaches have been applied in various applications, there is a lack of comparative studies on these approaches. Hence, there is a need to evaluate and compare different hybrid modeling patterns to determine their effectiveness.

## 3. Data and Methodology

This section provides an overview of the methodology and data utilized to establish hybrid models for predicting the final dimensions of the part based on the process settings. It begins by introducing the specimen, its material, and the simulated and experimental data. Next, an overview of the hybrid models used is given, followed by a detailed description of each approach, including the used domain knowledge and strategy for integrating data.

### 3.1. Specimen, Material, and Data Acquisition

**Figure 2:**
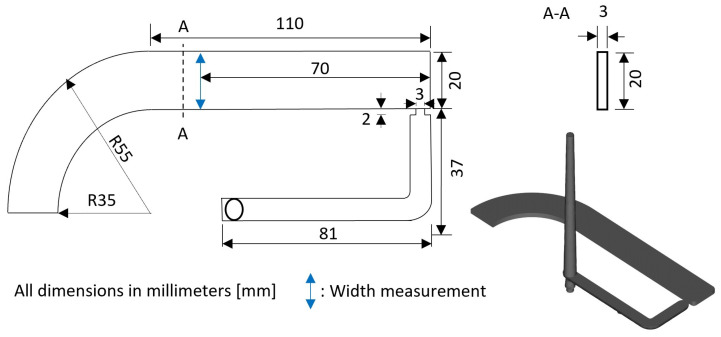
Illustration of the used part and measurement position.

A simple mold geometry is chosen for the evaluation of the hybrid approaches. The injection-molded part used in this work is shown in Figure 2. It is a thin-walled part characterized by a constant rectangular cross section. An unreinforced polyoxymethylene homo-polymer (POM) was chosen as the material due to its industrial relevance.

**Figure 3:**
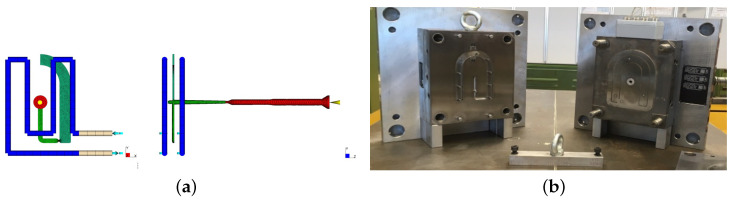
Simulation model and mold geometry used. (**a**) The meshed simulation model including the part (dark green), runner and sprue (light green), cooling channels (blue), and feed system (red) [21]. (**b**) The used mold for the experimental data acquisition.

The used simulation model is shown in Figure 3a and was already introduced in more detail in [26]. The simulations are carried out using Autodesk^®^ Moldflow^®^ Insight 2021.1 (AMI2021.1). For the material, the default parameter from the Moldflow database of the Material Delrin^®^ 111P NC010 from Delrin, Wilmington, DE, USA is selected. The 3D simulation includes filling, packing, and cooling phases.

The three process parameters $\theta =(T_{\mathrm{mold}},V_{\mathrm{inj}},P_{\mathrm{hold}})$ in Table 1 are varied in a full-factorial DoE across a total of 27 simulations. An overview of all combinations can be found in the Appendix A in Table A1. The process parameters are the coolant temperature $T_{c,\mathrm{in}}$, the injection velocity $V_{\mathrm{inj}}$ during the injection phase, and the holding pressure $P_{\mathrm{hold}}$. Instead of the actual controlled coolant temperature, the resulting approximated mold temperature $T_{\mathrm{mold}}=T_{c,\mathrm{in}}-4$ [26] is used.

The parameters $T_{\mathrm{mold}}$ and $V_{\mathrm{inj}}$ are chosen due to their influence on the temperature within the process. While the holding pressure does not impact the temperature significantly, it is the main influencing factor of the formed residual stresses $\sigma_{I_{ij}}$. An overview of the process settings, which are not varied, is presented in Table 2.

**Figure A1:**
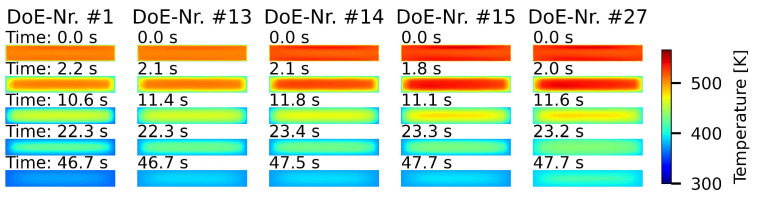
Examples of exported temperatures profiles within the cross section of the used part geometry at specific time steps of the simulation for different process parameter combinations. The network $N_{T}\left(t,x,\theta \right)$ is used to capture the dataset.

**Figure A2:**
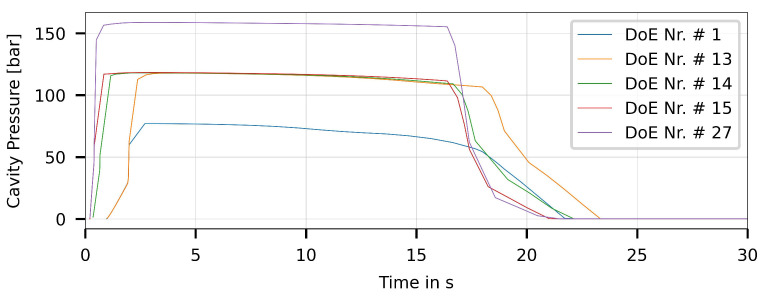
Examples of exported pressure profiles at the central node of the cross section of the used part geometry for different process parameter combinations. The network $N_{P}\left(t,x,\theta \right)$ is used to capture the dataset.

The data extracted from the simulation are the node values (at locations $x_{i}$ for a specific parameter combination $\theta_{i}$) of the pressure *P*, temperature *T*, and displacement *u*. Examples of the extracted temperature and pressure profiles of the simulated process are illustrated for various process settings in the Appendix A in Figure A1 and Figure A2. The displacement data u(x,θ) need to be transformed to the part width w(x,θ). For this, the displacements at the boundaries (1D case: $x_{\min}$, $x_{\max}$) are subtracted from the initial width $w_{0}=20$ mm of the geometry as follows:

(10) $$w\left(\theta \right)=w_{0}-\left(u\left(x_{\min},\theta \right)-u\left(x_{\max},\theta \right)\right)$$

For the experimental data generation, an electric injection molding machine (E-Motion 440/220 T from ENGEL AUSTRIA GmbH, Schwertberg, Austria) is used. The mold used is shown in Figure 3b. The drying of the material is achieved using a hot air dryer for 2 h at a temperature of 80 °C. To ensure comparable experimental conditions, the basic procedure is maintained for each experiment. In the experiment preparation, cylinder zone heating and mold temperature control are first switched on and left in this state for one hour to warm up without producing molded parts. After this waiting time, the production is started in a fully automatic mode, which is maintained for half an hour (30 parts). This ensures sufficient thermal equilibrium before the actual experiment is conducted according to the respective specifications. For each experimental setting, seven parts are produced fully automatically to capture any additional process variations. During the experiment, the parts are removed using a robot handling system. With 27 experimental settings, this results in 189 parts. A 3D profilometer (Model VR-5000 from Keyence Corporation, Osaka, Japan) is used to measure shrinkage in the width direction. The point of measurement can be seen in Figure 2. Depending on the type of measurement and the monitor magnification, this device has different accuracies. According to the manufacturer, it has an accuracy of ±5μm for width measurements at a 12× monitor magnification. For each process setting, the final widths in the experimental dataset are determined by calculating the average width across the seven repetitions. An overview of the process settings of the simulated and experimental average widths, along with the standard deviations across the seven repetitions, can be found in Table A1 in the Appendix A. The average standard deviation across the varied process settings is ±6.9μm, which is acceptably close to the measurement device’s accuracy.

### 3.2. Overview of the Hybrid Models and Training Data

The primary objective of the study is to compare hybrid modeling approaches with the aim of improving the predictive accuracy of data-driven models in low data regimes. The general modeling task is to predict the width *w*, which is dependent on the process settings θ. Surrogate modeling this type of dependency is the first step in injection molding optimization routines, as described in [5,16,17].

The literature showed that for these types of regression tasks in injection molding, NNs are a suitable choice due to their ability to model non-linear dependencies. Within this work, simple NNs with similar architectures are used to assess different hybrid approaches, aiming to physically inform the predictions while maintaining a consistent underlying data-based model to facilitate better comparability. NNs are additionally the choice of architecture since, due to their flexibility, different hybrid approaches can be used. For the implementation, the torchphysics library [27] was used, which uses pytorch [28] as a backend. The simulation dataset is utilized for the physics-based knowledge and to test the capabilities of the data-based, physics-based, and hybrid approaches. With the real measurement data, the hybrid approaches are further validated.

**Figure 4:**
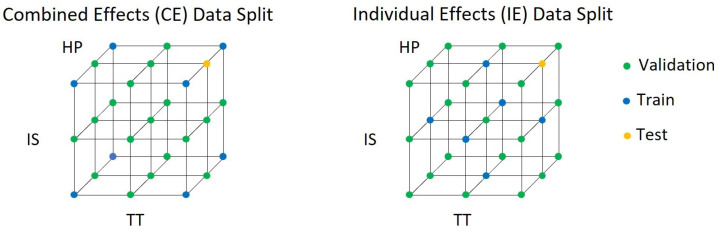
Used “Individual Effects” (IE) and “Combined Effects” (CE) data split.

The performance of the different hybrid models is evaluated using different sub-sections of the datasets. The models are trained on two different subsections labeled “Combined Effects (CE)” and “Individual Effects (IE)”, as shown in Figure 4. The CE data split is a Full-Factorial DoE with two factors (Low and High). The IE data split is a Star DoE. One additional parameter combination is used as a test setting for the hyperparameter optimization, as well as for an EarlyStopping [28] criteria. The remaining data points from the dataset are used as validation points. For both data splits, the training data is below 30% and contains only the linear effects of the process settings. By using the two data splits, it can be tested how well the hybrid approaches learn non-linear patterns incorporating a physics-based model. The mean absolute error (MAE) is used for the evaluation and comparison of the different approaches.

A 10-fold cross-validation is conducted since, due to the random initialization of the network weights and the random shuffling of the dataset, each optimization run of a network yields a slightly different result. The average across the 10 predictions is denoted by $MAE_{10}$.

In Table 3, an overview of the hybrid approaches is given. The first two models represent the purely data-based and purely physics-based approaches. The subsequent five models are the individual hybrid modeling patterns. The last five models are combinations of the individual hybrid modeling patterns with more complex models.

### 3.3. Base Models

#### 3.3.1. Data-Based Model

The data-based model serves as a baseline model, learning a direct relationship between the process settings θ and the part width *w*. With three process settings, the network has three inputs $\theta =(T_{\mathrm{mold}},V_{\mathrm{inj}},P_{\mathrm{hold}})$ and one output $w_{\mathrm{predicted}}$. The data loss $L_{\mathrm{Data}}$ used to train the network is modeled with the L2 norm of the error between the training data and the network predictions as follows:

(11) $$L_{\mathrm{Data}}=\frac{1}{N_{\mathrm{data}}}\sum_{i=1}^{N_{\mathrm{data}}}\left|\right|w_{\mathrm{data}}\left(\theta_{{\mathrm{data}}_{i}}\right)-w_{\mathrm{predicted}}\left(\theta_{{\mathrm{data}}_{i}}\right){\left|\right|}_{2}$$

Here, an individual process parameter combination is denoted by $\theta_{{\mathrm{Sim}}_{i}}$. ||·|| denotes the L2 norm.

To determine the optimal hyperparameters for the data-based approach, a grid search is conducted. Table 4 provides an overview of the hyperparameters and search space. The resulting optimal network architecture and optimization approach is highlighted within the following table.

The identified hyperparameters are maintained at a constant for the following data models within the hybrid approaches, unless specified otherwise. This promotes a better assessment of the impact of the various hybrid modeling patterns.

#### 3.3.2. Physics-Based Model

To physically predict the shrinkage behavior based on the process settings θ, the final displacement u(x,θ) needs to be determined. Here, the thermoelastic constitutive equation for the unidirectional composite (Equation (1)) is rewritten in terms of its strains as follows:

(12) $$\epsilon_{ij}^{\mathrm{total}}=\epsilon_{kl}^{\mathrm{th}}+\epsilon_{ij}$$

where the total strain $\epsilon_{ij}^{\mathrm{total}}$ is the sum of the elastic strain $\epsilon_{ij}$ and the thermal strain $\epsilon_{ij}^{\mathrm{thermal}}$. The thermal strain is expressed as follows [12]:

(13) $$\epsilon_{kl}^{\mathrm{th}}=\alpha \Delta T$$

where $\alpha =100e-6\frac{1}{{}^{\circ}K}$ represents the coefficient of linear thermal expansion (CTE) of the used polymer, taken from the CAMPUS^®^ (Computer-Aided Material Pre-selection by Uniform Standards) database, available at http://www.campusplastics.com/ (accessed on 10 July 2024). However, predicting the elastic strain $\epsilon_{ij}$ is challenging due to the complex formation of the residual stresses $\sigma_{I_{ij}}$ within the process. As a result, for the purely physics-based model, a simplification is made and only the thermal strains are considered. The modeling error is aimed to be compensated for utilizing the hybrid approaches.

In the reference simulation software, the thermal strain $\epsilon_{kl}^{\mathrm{th}}$ is calculated using the difference between the temperature at the end of the process $T_{\mathrm{end}}$ and room temperature $T_{\mathrm{room}}$. Instead of $T_{\mathrm{end}}$, in this work, the temperature $T_{P_{0}}$ at which the process reaches ambient pressure within the cavity and the part detaches from the wall is utilized. This temperature range is, e.g., used for calculating the volumetric shrinkage (see Section 2.1, Figure 1). Using this, parts of the temperature-dependent in-process shrinkage effects are included in the thermal strain.

To have $T_{P_{0}}$ available for different process settings, the entire dataset of the simulated temperatures and pressures is compressed into surrogate models, enabling interpolation to set combinations not covered by the dataset. NNs are trained for this purpose, with one NN for the temperature $N_{T_{\mathrm{Sim}}}\left(x,t,\theta \right)$ and another for the pressure $N_{P_{\mathrm{Sim}}}\left(x,t,\theta \right)$. Due to the fast inference time of the NN, the results can be obtained in real time. The network specifications for the surrogate models are in Table 5. The specifications were chosen according the networks’ ability to approximate the simulated temperatures and pressures, similarly to how it is described in [29]. Different network sizes were studied while monitoring the overall training, i.e., compression error. The same networks $N_{T_{\mathrm{Sim}}}\left(x,t,\theta \right)$ and $N_{P_{\mathrm{Sim}}}\left(x,t,\theta \right)$ are used for the physics-based as well as hybrid models, increasing the comparability of the different approaches by sourcing the same information.

Once trained, the surrogates can then be used to calculate $T_{P_{0}}\left(x,\theta \right)$. For a given setting combination $\theta_{i}$ at a specific location $x_{i}$, the model $N_{P_{\mathrm{Sim}}}\left(t,x_{i},\theta_{i}\right)$ is utilized to predict the pressure at n=100 discrete time points on a set interval from $t_{\mathrm{start}}$ to $t_{\mathrm{end}}$. Subsequently, the time point $t_{P_{0}}$ at which the predicted pressure drops below a threshold ε=1+0.1 bar is determined ($N_{P_{\mathrm{Sim}}}\left(t_{j},x_{i},\theta_{i}\right)<\varepsilon \to t_{j}\approx t_{P_{0}}$), and this value is used to predict the temperature $T_{P_{0}}\left(x_{i},\theta_{i}\right)$ using $N_{T_{\mathrm{Sim}}}\left(t_{j},x_{i},\theta_{i}\right)$.

To calculate the resulting part width, a 1D simplification is used. By employing the constitutive strain–displacement relationship (Equation (4)), the final displacement can be obtained using integration as follows:

(14) $$u_{x}\left(x,\theta \right)=\int \epsilon_{kl}^{\mathrm{th}}\left(x,\theta \right)\;dx$$

In this work, a standard integration scheme is implemented with $u_{x_{0}}\left(x_{0}=0\right)=0$ and the following:

(15) $$u_{x_{i}}=\left(x_{i}-x_{i-1}\right)\epsilon_{kl}^{\mathrm{th}}\left(x_{i},\theta \right)+u_{x_{i-1}}$$

The final width of the part can be calculated by taking the final displacement of the boundary points $x_{\min}=0\;$mm and $x_{\max}=20\;$mm and subtracting it from the original geometry, where the original part width is $w_{0}=20\;$mm:

(16) $$w\left(\theta \right)=P\left(\theta \right)=w_{0}-\int_{x_{\min}}^{x_{\max}}\epsilon_{kl}^{\mathrm{th}}\left(x,\theta \right)\;dx$$

### 3.4. Hybrid Models

#### 3.4.1. FL

Using the FL approach, a calibration of the physics-based model is targeted. Detailed domain knowledge about the shortcomings of the used physics-based model is necessary to ensure robust extrapolation capabilities. In the used physics-based model, the temperature-dependent shrinkage effects have been addressed, but the elastic strain $\epsilon_{ij}$ occurring due to residual stresses $\sigma_{I_{ij}}$, formed during the processes, has not been considered (Equations (1) and (12)). While for non-reinforced thermoplastic materials, the thermal strain accounts for the main effects of the tool temperature and injection velocity, the influence of pressure remains a challenge for physics-based models as well as for most standard simulation softwares. Hence, with the FL approach, a data-driven relationship between the elastic strain $\epsilon_{ij}\left(P_{hold}\right)$ and the holding pressure is modeled. Instead of a NN, for the FL approach, a linear regression model (y=ax+b) is chosen to model $\epsilon_{ij}\left(P_{hold}\right)$ due to the limited training samples in the dataset for the chosen input parameter $P_{hold}$. For the IE data split, only two observations of variations in $P_{hold}$ are found within the training dataset, making a linear regression model with two degrees of freedom an optimal choice. To estimate the coefficients, combinations of process parameters from the training data that show variations at lower pressures $\theta_{P_{{hold}_{{-}_{j}}}}$ and higher pressures $\theta_{P_{{hold}_{{+}_{k}}}}$ are used to calculate the minimum and maximum total effective strains $\epsilon_{P_{{hold}_{-}}}$ and $\epsilon_{P_{{hold}_{+}}}$, respectively:

(17) $$\epsilon_{P_{{hold}_{-}}}=\frac{1}{n_{data_{-}}}\sum_{j=0}^{n_{data_{-}}}\frac{w_{\mathrm{data}}\left(\theta_{P_{{hold}_{{-}_{j}}}}\right)-w_{0}}{w_{0}}$$

(18) $$\epsilon_{P_{{hold}_{+}}}=\frac{1}{n_{data_{+}}}\sum_{k=0}^{n_{data_{+}}}\frac{w_{\mathrm{data}}\left(\theta_{P_{{hold}_{{+}_{k}}}}\right)-w_{0}}{w_{0}}$$

Using the fittet total effective strains $\epsilon_{P_{{hold}_{-}}}$ and $\epsilon_{P_{{hold}_{+}}}$, the linear model is given by the following:

(19) $$\epsilon_{ij}\left(P_{hold}\right)=\frac{\left(P_{hold}-P_{{hold}_{-}}\right)}{\left(P_{{hold}_{+}}-P_{{hold}_{-}}\right)}\left(\epsilon_{P_{{hold}_{+}}}-\epsilon_{P_{{hold}_{-}}}\right)+\epsilon_{P_{{hold}_{-}}}$$

The corrected displacement values are calculated using the same integration scheme, incorporating the estimated elastic strain as follows:

(20) $$u_{\mathrm{corrected}}\left(x_{i}\right)=\left(x_{i}-x_{\left(i-1\right)}\right)\left(\epsilon_{kl}^{th}\left(x_{i},\theta \right)+\epsilon_{ij}\left(P_{hold}\right)\right)+u\left(x_{\left(i-1\right)}\right)$$

The resulting part width is then determined using the corrected displacement values as follows:

(21) $$w\left(\theta \right)=P_{D}\left(\theta \right)=l-\int_{x_{\min}}^{x_{\max}}\epsilon_{kl}^{th}\left(x,\theta \right)+\epsilon_{ij}\left(P_{hold}\right)dx$$

#### 3.4.2. DM

The DM approach builds on the physics-based model by using the same inputs as the purely data-based approach, but focuses on predicting the deviation from the physics-based model. By subtracting the physics-based model $P(\theta_{\mathrm{data}})$ from the observed data $w_{\mathrm{data}}$, one obtains the delta which is learned by the delta model using a NN.

#### 3.4.3. DM + FL

The extension for the DM approach is the utilization of the calibrated physics-based model via the FL approach. Within this hybrid approach, the data are used to calibrate the physics model, as well as to learn the differences between the physics model and the observed data points.

#### 3.4.4. FT

For the FT approach, first, a NN is pretrained on a separate dataset created by the physical model P(θ). The full-factorial DoE with three steps (Table 1) is used to create discrete predictions by the physics-based model. The actual training data are then used to fine-tune the pretrained model. The model uses the same inputs as a purely data-driven model and directly estimates the resulting part width. Notably, this approach does not involve freezing any layers during fine-tuning (i.e., transfer learning) as initial studies indicated that doing so does not enhance performance for the shallow networks employed.

#### 3.4.5. FT + FL

This extension of the FT method involves pretraining the NN on a dataset specifically generated through the FL approach. This step aims to leverage the refined data insights from FL for an even more accurate model initialization before fine-tuning with actual training data.

#### 3.4.6. PP

For the PP approach, the prediction of the physics-based model serves as an additional input feature of the NN. This means that the network has four input parameters, the three process settings as well as the physics-based prediction P(θ). The output of the network is the prediction of the final width.

#### 3.4.7. PP + FL

Like in the previous hybrid approaches, the combination of the PP approach with the FL approach is created by substituting the standard physics-based prediction P(θ) with the enhanced prediction $P_{D}\left(\theta \right)$ from the FL approach.

#### 3.4.8. PC

Within this work, a soft constraint approach is examined, using conditional PINNs [30]. PINNs have the ability to incorporate spatiotemporal ODEs and PDEs into the learning process using additional physics-based loss terms [31]. Due to the general applicability of differential descriptions within the engineering domain, the approach has been used to study, e.g., heat equations [32], flow equations [33], and solid mechanics [29]. In [22,30], a more detailed description of the PINN methodology for solving parameterized ODEs and PDEs is given.

For the implementation of the physics-based shrinkage behavior with PINNs, the complexity of the data-based model is increased by adding a 1D spatial domain. The input parameters of the NN include the location parameter *x* next to the process parameters θ. Instead of predicting the resulting part width directly, the displacement $u_{PC}\left(x,\theta \right)$ is modeled like in the physics-based approach. The final width can be analytically obtained again by using the predicted displacement $u_{PC}\left(x,\theta \right)$. The data-based losses as well as the physics-based losses for the displacement are introduced in the following.

##### Data-Based Loss

Since the network predicts the displacement, the resulting part width must be transformed into displacement data. While the final displacements are available for the simulation dataset, for the real parts, only the resulting part width is measured. To generate displacement data from width measurements, a simple linear displacement is assumed, starting from a zero-displacement point $x_{0}$, like in the physics-based model. The effective strain is calculated as follows:

(22) $$\epsilon_{\mathrm{eff}}\left(\theta \right)=\frac{w_{0}-w_{\mathrm{data}}\left(\theta \right)}{w_{0}}$$

Using the strain–displacement relationship, displacement data $u_{\mathrm{data}}\left(x,\theta \right)$ are derived for both the minimum ($x_{\min}$) and maximum ($x_{\max}$) values of the 1D domain:

(23) $$u_{\mathrm{data}}\left(x_{\min},\theta \right)=\left(x_{0}-x_{\min}\right)\epsilon_{\mathrm{eff}}\left(\theta \right)$$

(24) $$u_{\mathrm{data}}\left(x_{\max},\theta \right)=\left(x_{0}-x_{\max}\right)\epsilon_{\mathrm{eff}}\left(\theta \right)$$

The data-based loss is defined as follows:

(25) $$L_{Data_{PC}}=\frac{1}{N_{Data_{PC}}}\sum_{i=1}^{N_{Data_{PC}}}\left|\right|u_{PC}\left(x_{i},\theta_{i}\right)-u_{\mathrm{data}}\left(x_{i},\theta_{i}\right)\left|\right|$$

The number of training points, in this case $N_{Data_{PC}}=N_{\theta_{\mathrm{train}}}N_{x_{\mathrm{train}}}$, are the number of training settings $N_{\theta_{\mathrm{train}}}$ multiplied by the number of observed (boundary) points $N_{x_{\mathrm{train}}}=2$.

##### Physics-Based Loss

The network architecture is chosen such that by using automatic differentiation, the gradient $\frac{du_{PC}\left(x,\theta \right)}{dx}$ can be obtained for creating a physics-based loss [34]. The physical loss uses a soft constraint, as well as the same physical process description and pretrained temperature and pressure models from Section 3.3.2, to obtain the total strain $\epsilon_{ij}^{\mathrm{total}}\left(\theta \right)$ during the training process:

(26) $$L_{\epsilon }=\frac{1}{N_{\epsilon }}\sum_{i=1}^{N_{\epsilon }}\left(\frac{du_{PC}\left(x,\theta \right)}{dx}-\epsilon_{ij}^{\mathrm{total}}\left(\theta \right)\right)$$

An additional loss enforces a zero-displacement condition at the point $x_{0}$ across the input domain of the process parameters θ:

(27) $$L_{x_{0}}=\frac{1}{N_{x_{0}}}\sum_{i=1}^{N_{x_{0}}}u_{PC}\left(x=x_{0},\theta \right)$$

While the observed points of the data-based loss are only on the boundary, the physics-based loss can be applied across the whole input domain. Specifically, the domain for $L_{\epsilon }$, denoted as $\Omega_{\epsilon }$, spans both the process parameter space $\Omega_{\theta }$ and the spatial domain $\Omega_{x}$, whereas the loss enforcing zero displacement $L_{x_{0}}$ operates over $\Omega_{\theta }$ and at a specific spatial location $x_{0}$. The process parameter domain $\Omega_{\theta }$ is within the range of values of the used full-factorial DoE (Table 1). The spatial domain is $\Omega_{x}=\left[0\;\mathrm{mm},20\;\mathrm{mm}\right]$, the width of the original geometry. To generate data points for evaluating the physics-based loss, a random uniform sampling strategy is employed. The number of points $\left(\theta_{i},x_{i}\right)$ sampled at each training step are as follows: $N_{\epsilon }=N_{x_{0}}=1200$.

The optimization of PINNs relies on a loss function that is a weighted sum of the data-based loss $L_{Data_{PC}}$, the zero-displacement loss $L_{x_{0}}$, and the physics-based loss $L_{\epsilon }$ [31]:

(28) $$L=w_{Data_{PC}}L_{Data_{PC}}+w_{x_{0}}L_{x_{0}}+w_{\epsilon }L_{\epsilon }$$

The weighting factors for these losses can be adjusted to prioritize certain aspects of the model. In this study, the weights $w_{x_{0}}$ and $w_{Data_{PC}}$ are set to 1, while the weight $w_{\epsilon }$ is significantly higher at 100. This higher weighting for $L_{\epsilon }$ is chosen to balance the scale of the loss terms, considering that the magnitude of strain values within $L_{\epsilon }$ is generally smaller than that of the overall displacement values. This strategic weighting ensures that each aspect of the loss function contributes appropriately to the model’s training, aiming for an accurate and physically consistent prediction of the system behavior.

#### 3.4.9. PC + FL

For the extension of the PC approach with the FL approach, the corrected total strain $\epsilon_{kl}^{th}\left(x_{i},\theta \right)+\epsilon_{ij}\left(P_{hold}\right)$ (Equation (12)) is used within the physics-based loss function $L_{\epsilon }$ (Equation (26)). This adjustment allows the model to incorporate more accurate physical constraints derived from both theoretical and empirical insights, enhancing the model’s predictive accuracy.

#### 3.4.10. PC + FT + FL

This approach leverages the 1D displacement calculations from the physics-based model to pretrain the NN. By utilizing the detailed displacement data, the network gains an initial understanding of the physical dynamics. The final hybrid model emerges from fine-tuning this pretrained network with the additional physical constraints and observed data, creating a model that benefits from both the depth of physics-based analysis and the adaptability of ML techniques.

## 4. Results

In this chapter, the results of the different hybrid approaches are presented, assessing their performance across different datasets (Simulated and Experimental) and data splits (IE and CE). First, the results of the purely data- and physics-based approach are shown and discussed, after which the results of the hybrid approaches are compared.

### 4.1. Base Models

**Figure 5:**
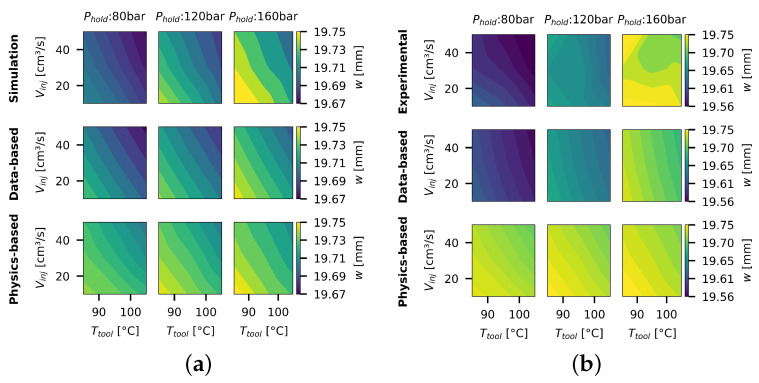
Sampled data points for the inverse approach. (**a**) Contour plots of the simulated widths *w* dependent on process settings θ, where the data-based model has been trained on the IE data split. (**b**) Contour plots of the experimental widths *w* dependent on process settings θ, where the data-based model has been trained on the IE data split.

For a first comparison of the base models, contour plots are created through the linear interpolation of the part width at the process settings of the full-factorial DoE with three levels. These plots are shown in Figure 5a for the simulation dataset and in Figure 5b for the experimental dataset.

Firstly, looking at the differences of the simulated and experimental datasets, the increased complexity of the real process behavior is visible. This is also reflected within the $MAE_{10}^{total}$, where the data-based model can fit the IE data split with an $MAE_{10}^{total}$ of 0.0083 mm on the simulated dataset, while on the experimental dataset, the $MAE_{10}^{total}$ increases to 0.0168 mm. Importantly, the average standard deviation observed in the experimental width measurements (±0.0069 mm) indicates the potential lowest MAE attainable for the experimental dataset. Given that the data splits only encompass linear effects, the purely data-driven model’s predictions are inherently linear.

While the simplified physics-based model demonstrates its capability to predict the directionality of effects for $T_{\mathrm{mold}}$ and $V_{inj}$, an overall offset can be observed. This offset is more pronounced at lower $P_{hold}$, suggesting that the model’s inability to account for residual stresses contributes to its discrepancies. While the model aligns well with the simulated effects of $T_{\mathrm{mold}}$ and $V_{inj}$, it fails to capture the non-linear dynamics present in the real process, particularly under varying $P_{hold}$.

Following this, the results of the hybrid approaches are shown, first on the simulated dataset and then on the experimental dataset.

### 4.2. Hybrid Approaches

#### 4.2.1. Simulated Dataset

The summarized results in Table 6 highlight the train $MAE_{10}^{train}$, validation $MAE_{10}^{val}$, and total $MAE_{10}^{total}$ across different models and data splits, offering a quantitative basis for assessing the performance of each hybrid approach.

The analysis reveals a clear trend: hybrid modeling patterns mostly achieve a better accuracy and lower standard deviation than the purely data-based model. This improvement underscores the significant advantage of incorporating physics-based insights into ML models, enhancing their predictive accuracy, robustness, and generalization capabilities.

##### Feature Learning (FL)

The FL approach shows an improvement in accuracy over the purely physics-based model, with an IE $MAE_{10}^{total}$ of 0.0054 mm and a CE $MAE_{10}^{total}$ of 0.0057 mm. Since the final prediction of the FL approach is physics-based, the standard deviation is 0.0. This indicates the effectiveness of integrating data-based features, which enables the physics-based model to better capture the underlying dynamics of the injection molding process, thereby reducing prediction errors. However, since only the variations of the holding pressure are captured, the purely data-based model outperforms the FL approach using the CE data split. On the other hand, with only two examples, the physics-based model is able to extrapolate the unobserved $T_{\mathrm{mold}}$ and $V_{\mathrm{inj}}$ variations with good accuracy within the IE data split.

##### Delta Model (DM) and Fine-Tuning (FT)

Both DM and FT approaches exhibit promising performances, with the FT approach being slightly better with a CE $MAE_{10}^{total}$ of 0.0027 mm compared to the DM’s CE $MAE_{10}$ of 0.0029 mm. Across both data splits, the combined FT+FL approach achieves the lowest validation $MAE_{10}^{val}$.

##### Physics-Based Preprocessing (PP)

The PP approach, while not achieving the top performance, still improves upon the purely data-based model with a CE $MAE_{10}^{total}$ of 0.0031 mm. This highlights the benefit of augmenting the input feature space with physics-based predictions, providing the model with additional contextual information for more accurate predictions.

##### Physical Constraints (PC)

Remarkably, the PC approach, especially when combined with FL (PC + FL), achieves one of the lowest $MAE_{10}^{total}$, with a CE $MAE_{10}^{total}$ of 0.0024 mm. The PC approaches notably achieve the lowest $MAE_{10}^{train}$. This superior performance is likely due to the direct integration of physical laws as regularization terms, ensuring that predictions not only adhere to known physical constraints but also align closely with the observed data. In contrast, while the L2 regularization employed in the other approaches is pivotal for robust generalization, it results in a higher $MAE_{10}^{train}$.

##### Combined Hybrid Approach

The combined hybrid model PC + FL + FT stands out with an impressive CE $MAE_{10}^{total}$ of 0.0022 mm, showcasing the potential of leveraging multiple hybrid modeling patterns for enhanced accuracy. This approach, by integrating feature learning, fine-tuning, and physical constraints, effectively captures the complex dynamics of the injection molding process, resulting in the most accurate surrogate model for the CE data split.

The comparative analysis of hybrid ML approaches on the simulated dataset demonstrates the clear benefits of integrating physics-based knowledge with data-driven models. The combined hybrid models, particularly PC + FL + FT, emerge as the most effective in accurately capturing part shrinkage in injection molding processes. While each hybrid approach uses the same underlying physics-based model, combining different strategies of extracting knowledge can lead to superior results. Concerning the overall accuracy, already with the IE data split, the MAE, using a hybrid modeling approach, is able to surpass the average repetition accuracy of the experimental process ±0.0069 mm. By utilizing more accurate and data-efficient underlying surrogate models, this approach enhances the potential for improved optimization tasks in the injection molding process, as, e.g., illustrated in [4].

#### 4.2.2. Experimental Dataset

In this subsection, the hybrid ML approaches are further examined using the experimental dataset to gain a more nuanced understanding of their performance in real-world scenarios. The results, shown in Table 7, offer a granular view of the predictive accuracy of each model across two different data splits. This analysis is instrumental in showcasing the practical applicability of these hybrid models in the domain of injection molding, particularly for the prediction of part shrinkage.

The analysis of the experimental dataset reveals a complex landscape of model performance, with hybrid approaches generally demonstrating enhanced predictive capabilities over purely data-driven and physics-based models. The data-based model exhibits an IE $MAE_{10}^{total}$ of 0.0146 mm and a CE $MAE_{10}^{total}$ of 0.0124 mm, setting a baseline for the evaluation of hybrid models. On the other hand, the average standard deviation of ±0.0069 mm sets the benchmark for an optimal MAE with the attained experimental measurements.

##### Feature Learning (FL)

The FL approach is showing the same $MAE_{10}^{total}$ in the IE data split (0.0114 mm) and the CE data split (0.0114 mm). For the CE data split, this achieves the best validation $MAE_{10}^{val}$, showcasing the capability of the calibrated physics-based model to predict the effects of $T_{\mathrm{mold}}$ and $V_{inj}$ also in real applications. The robust extrapolation capabilities, especially for the IE data split, is supported by the mostly linear dependency between $P_{hold}$ and the width *w* in the given design space. However, the used linear regession model is not flexible enough to fit non-linear relationships, which is shown in a comparably high $MAE_{10}^{train}$.

##### Delta Model (DM) and Fine-Tuning (FT)

Both the DM and FT approaches show a good performance on the experimental dataset, with the FT approach marginally outperforming the DM in the CE data split with an $MAE_{10}^{total}$ of 0.0111 mm. This suggests that the strategy of leveraging pretraining on physics-based predictions, followed by fine-tuning with empirical data, is beneficial in real-world applications. A combination with the FL approach achieves for both hybrid modelling patterns a further improvement in prediction accuracy.

##### Physics-Based Preprocessing (PP)

The PP approach, despite its integration of physics-based predictions as input features, yields a CE $MAE_{10}^{total}$ of 0.0124 mm, mirroring the baseline set by the purely data-driven model. However, a lower standard variation indicates an improved robustness. The combined approach, PP + FL, shows enhanced robustness and higher prediction accuracy compared to the data-driven model.

##### Physical Constraints (PC)

The PC approach, particularly in its standalone form, encounters challenges, as shown by a CE $MAE_{10}^{total}$ of 0.0276 mm. However, when combined with FL (PC + FL), it demonstrates a marked improvement, achieving a CE total $MAE_{10}$ of 0.0111 mm and the best performance on the IE data split with a $MAE_{10}^{total}$ of 0.0104 mm. Furthermore, it demonstrates a comparable or lower standard deviation than the purely data-driven approach, thereby enhancing its robustness. This improvement illustrates the value of embedding physical laws directly into the learning process, especially when calibrated with data-driven insights.

##### Combined Hybrid Approach

The combined hybrid model PC + FL + FT exhibits a slightly worse performance than PC + FL, with a CE $MAE_{10}^{total}$ of 0.0121 mm. The results indicate that adding more hybrid modeling patterns does not always improve the model. While various hybrid models can extract information, the total amount of information in the data and physics is finite, creating a potential upper limit. Nevertheless, leveraging the unique strengths of different hybrid modeling patterns improves the robustness in the studied case, with room for further optimization.

Overall, the examined hybrid modeling approaches showed an improved and more robust prediction accuracy for the real injection molding process for both data splits. For the IE data split, having less training data available, the improvements achieved by adding physical knowledge has a higher effect. While with the models trained on simulation data, the repetition accuracy of the measurement device could be surpassed, the increased non-linear behavior within the real process increases the overall MAE for all approaches. However, the achieved accuracy of the surrogate model is already close to the measurement accuracy of the device at hand.

## 5. Conclusions and Outlook

In this work, a comprehensive exploration of hybrid ML approaches for the surrogate modeling of part shrinkage in injection molding processes has been carried out. These types of surrogate models are at the basis of most optimization workflows for injection molding. By increasing the overall accuracy and data efficiency of surrogate models using hybrid modeling patterns, this consequently contributes to enhanced subsequent optimization routines.

By comparing five distinct individual hybrid modeling patterns and developing more complex hybrid strategies systematically, the potential of integrating physics-based knowledge with data-driven ML models to enhance predictive accuracy and generalizability has been shown. The used formalization of these hybrid modeling patterns provides a clear framework for understanding and combining the different approaches.

The comparative analysis across both simulated and experimental datasets revealed that hybrid approaches generally outperform purely data-driven and physics-based models, with a higher relative improvement in terms of accuracy observed for the smaller IE data split. Notably, the FL approach, DM, and FT strategies emerged as particularly effective, with the FT approach showing a marked improvement in predictive accuracy, especially in the CE data split. This underscores the value of pretraining models on physics-based predictions before refining them with empirical data, allowing for a foundational understanding of process physics that is enhanced through exposure to real-world data. The FL approach proved to be the most efficient, particularly for the IE data split, where it could extrapolate to different temperature and injection speed settings without any reference data.

The PC approach, especially when combined with FL as PC + FL, showcased a robust capability to navigate the complexities inherent in experimental data, achieving one of the lowest MAE across both data splits. This illustrates the effectiveness of embedding physical laws directly into the learning process that is calibrated with data-driven insights. More complex hybrid models, combining multiple hybrid modeling patterns, outperformed the individual approaches in most cases, with the FT + FL approach having the best average performance and the more complex PC + FL approach performing best on the real dataset. The exploration of further combined hybrid patterns, particularly PC + FL + FT, highlighted the potential of leveraging multiple hybrid modeling patterns.

This investigation into hybrid ML approaches for surrogate modeling in injection molding has laid a foundation for future research aimed at optimizing these models for greater efficiency and integrating them in process optimization tasks. Individual research must continue in the fields of improving purely data-based as well as physics-based models. A further development of the physics-based models is necessary for a more detailed integration of the underlying physical principals. For example, the utilized strain–displacement relationship could be extended to integrate the spatiotemporal stress–strain-based process description. However, the non-linear process behavior of the real process, not covered by the physics-based model, only can be resolved by adding additional observations into the training dataset. Consequently, further investigations into the optimal amount of training data, along with a selection strategy between the different hybrid modeling approaches, need to be completed for an optimal utilization of the underlying physics- and data-based knowledge. Combining hybrid modeling techniques with dynamic DoE approaches, e.g., active learning, could further enhance the models’ predictive capabilities while keeping the necessary resources to a minimum. Possible future extensions include the integration of additional material, process, and geometric parameters, as well as predicting further process and quality attributes. This would enhance their applicability for subsequent optimization tasks, thus offering a more comprehensive approach to optimizing processes, materials, and designs for polymer products. As these hybrid models are improved, it is expected that they will play a key role in creating digital twins of the injection molding processes, ultimately contributing to the optimization and production of higher quality parts with reduced time and cost implications.

## Figures and Tables

**Table 1:** Steps used in the full-factorial DoE.

θ	$T_{\mathrm{mold}}$ (°C)	$V_{\mathrm{inj}}$ (${\mathrm{cm}}^{3}$/s)	$P_{\mathrm{hold}}$ (MPa)
+	85	10	80
0	95	30	120
−	105	50	160

**Table 2:** Fixed process settings.

Parameter	Value
Holding Time	16 s
Cooling Time	30 s
Melt Temperature	215 °C
Switch-Over	10 ${\mathrm{cm}}^{3}$

**Table 3:** Models used in the study and their corresponding formalization.

Model	Formalization
Data-based	w=D(θ)
Physics-based	w=P(θ)
FL	$w=P_{D}\left(\theta \right)$
DM	w=D(θ)+P(θ)
FT	w=D(θ)←P(θ)
PP	w=D(θ,P(θ))
PC	$w=D_{P}\left(\theta \right)$
DM + FL	$w=D\left(\theta \right)+P_{D}\left(\theta \right)$
FT + FL	$w=D\left(\theta \right)\leftarrow P_{D}\left(\theta \right)$
PP + FL	$w=D(\theta ,P_{D}\left(\theta \right))$
PC + FL	$w=D_{P_{D}}\left(\theta \right)$
PC + FT + FL	$w=D_{P_{D}}\left(\theta \right)\leftarrow P_{D}\left(\theta \right)$

**Table 4:** Hyperparameters and search space for the data-based model, where the resulting optimal network architecture and optimization approach are highlighted.

Parameter	Values
Network Size	[5,5], [10,10], **[20,20]**
L2 Regularization	0.05, **0.005**, 0.00001
Activation Function	ReLU(), **Tanh()**
Optimizer	**Adam**, SGF
Learning Rate	1 × $10^{-3}$, **5** × **10^−4^**, 1 × $10^{-4}$, 1 × $10^{-5}$

**Table 5:** Network specifications for the surrogate models.

Parameter	Values
Network Size	[80,80,80,80]
Activation Function	Tanh()
Optimizer	Adam
Learning Rate	1 × $10^{-3}$

**Table 6:** Model performance comparison for the simulated dataset across the 10-fold cross validation.

Model	IE			CE		
	$MAE_{10}^{train}$ [mm]	$MAE_{10}^{val}$ [mm]	$MAE_{10}^{total}$ [mm]	$MAE_{10}^{train}$ [mm]	$MAE_{10}^{val}$ [mm]	$MAE_{10}^{total}$ [mm]
Data-Based	0.0035	0.0083	0.0072	0.0041	0.0029	0.0033
	±0.0009	±0.0015	±0.0014	±0.0014	±0.0024	±0.0015
Physics-Based	0.0185	0.0209	0.0204	0.0223	0.0195	0.0204
	±0.0	±0.0	±0.0	±0.0	±0.0	±0.0
FL	0.0029	0.0062	0.0054	0.0072	0.0051	0.0057
	±0.0	±0.0	±0.0	±0.0	±0.0	±0.0
DM	0.0025	0.0064	0.0056	0.0038	0.0026	0.0029
	±0.0006	±0.0009	±0.0008	±0.0012	±0.0007	±0.0008
FT	0.0024	0.0062	0.0053	0.0030	0.0026	0.0027
	±0.0009	±0.0011	±0.0009	±0.0011	±0.0011	±0.0009
PP	0.0035	0.0080	0.0070	0.0046	0.0025	0.0031
	±0.0018	±0.0025	±0.0023	±0.001	±0.0005	±0.0006
PC	0.0005	0.0059	0.0047	0.0070	0.0023	0.0037
	±0.0001	±0.0043	±0.0033	±0.0066	±0.002	±0.0033
DM + FL	0.0014	0.0053	0.0045	0.0046	0.0030	0.0035
	±0.0004	±0.0028	±0.0023	±0.0008	±0.0036	±0.0027
FT + FL	0.0025	0.0041	0.0038	0.0041	0.0023	0.0028
	±0.0016	±0.0019	±0.0018	±0.0014	±0.0012	±0.0012
PP + FL	0.0028	0.0073	0.0063	0.0052	0.0028	0.0035
	±0.0013	±0.0019	±0.0018	±0.0006	±0.0003	±0.0004
PC + FL	0.0014	0.0048	0.0041	0.0013	0.0028	0.0024
	±0.0004	±0.0005	±0.0005	±0.0012	±0.001	±0.0006
PC + FL + FT	0.0016	0.0056	0.0047	0.0013	0.0026	0.0022
	±0.0005	±0.0007	±0.0004	±0.0002	±0.0002	±0.0002

**Table 7:** Model performance comparison for the simulated dataset across the 10-fold cross validation.

Model	IE			CE		
	$MAE_{10}^{train}$ [mm]	$MAE_{10}^{val}$ [mm]	$MAE_{10}^{total}$ [mm]	$MAE_{10}^{train}$ [mm]	$MAE_{10}^{val}$ [mm]	$MAE_{10}^{total}$ [mm]
Data-Based	0.0068	0.0168	0.0146	0.0082	0.0141	0.0124
	±0.0012	±0.0012	±0.0012	±0.0024	±0.003	±0.0017
Physics-Based	0.0805	0.0774	0.0781	0.0782	0.0781	0.0781
	±0.0	±0.0	±0.0	±0.0	±0.0	±0.0
FL	0.0055	0.0131	0.0114	0.0186	0.0084	0.0114
	±0.0	±0.0	±0.0	±0.0	±0.0	±0.0
DM	0.0014	0.0015	0.0012	0.0093	0.0154	0.0136
	±0.0012	±0.0015	±0.0014	±0.0026	±0.0023	±0.0019
FT	0.0045	0.0154	0.0130	0.0069	0.0128	0.0111
	±0.0016	±0.0027	±0.0022	±0.0024	±0.0039	±0.0028
PP	0.0061	0.0165	0.0142	0.0088	0.0140	0.0124
	±0.0006	±0.0006	±0.0005	±0.0029	±0.0012	±0.0013
PC	0.0007	0.0198	0.0156	0.0008	0.0389	0.0276
	±0.0003	±0.0088	±0.0068	±0.001	±0.008	±0.0057
DM + FL	0.0052	0.0138	0.0119	0.0110	0.0133	0.0127
	±0.0002	±0.0003	±0.0003	±0.0036	±0.0018	±0.002
FT + FL	0.0051	0.0139	0.0119	0.0103	0.0132	0.0124
	±0.0004	±0.0006	±0.0004	±0.0027	±0.0014	±0.0017
PP + FL	0.0059	0.0164	0.0141	0.0084	0.0128	0.0115
	±0.0016	±0.0019	±0.0018	±0.0026	±0.0015	±0.0013
PC + FL	0.0032	0.0124	0.0104	0.0016	0.0151	0.0111
	±0.0015	±0.0015	±0.0012	±0.0009	±0.0012	±0.001
PC + FL + FT	0.0045	0.0138	0.0117	0.0027	0.0161	0.0121
	±0.0018	±0.0022	±0.0015	±0.0013	±0.0011	±0.0008

## Data Availability

Datasets generated during the current study are available from the corresponding author on reasonable request.

## Abbreviations

The following abbreviations are used in this manuscript:

NN	Neural Network
ML	Machine Learning
PINN	Physics-Informed Neural Network
RSM	Response Surface Method
ANOVA	Analysis of Variance
DoE	Design of Experiments
POM	Polyoxymethylene
CTE	Coefficient of Thermal Expansion
FT	Fine-Tuning
PP	Preprocessing
PC	Physical Constraints
DM	Delta Model
FL	Feature Learning
CE	Combined Effects
IE	Individual Effects

## Appendix A

**Table A1:** Used DoE with simulated and experimental measured resulting part widths.

$T_{cool}\;\left[{}^{\circ}C\right]$	$P_{hold}\;\left[\mathrm{MPa}\right]$	$V_{inj}\;\left[\frac{{\mathrm{cmm}}^{3}}{s}\right]$	DoE Nr.	Sim. Width [mm]	Exp. Width ± Std. [mm]
84	20	10	1	19.695	19.615 ± 0.0017
84	20	30	2	19.692	19.591 ± 0.0023
84	20	50	3	19.691	19.586 ± 0.0021
84	50	10	4	19.705	19.668 ± 0.0021
84	50	30	5	19.704	19.653 ± 0.004
84	50	50	6	19.702	19.6785 ± 0.0029
84	80	10	7	19.721	19.751 ± 0.0005
84	80	30	8	19.715	19.724 ± 0.0103
84	80	50	9	19.713	19.786 ± 0.0167
99	20	10	10	19.694	19.603 ± 0.001
99	20	30	11	19.691	19.581 ± 0.0016
99	20	50	12	19.689	19.568 ± 0.0063
99	50	10	13	19.702	19.659 ± 0.01
99	50	30	14	19.700	19.659 ± 0.0025
99	50	50	15	19.697	19.654 ± 0.0026
99	80	10	16	19.717	19.7545 ± 0.0143
99	80	30	17	19.711	19.713 ± 0.0127
99	80	50	18	19.707	19.713 ± 0.0168
114	20	10	19	19.689	19.576 ± 0.0034
114	20	30	20	19.684	19.557 ± 0.0034
114	20	50	21	19.681	19.55 ± 0.0053
114	50	10	22	19.696	19.638 ± 0.0056
114	50	30	23	19.691	19.623 ± 0.0031
114	50	50	24	19.686	19.617 ± 0.0052
114	80	10	25	19.705	19.739 ± 0.0171
114	80	30	26	19.697	19.7135 ± 0.0203
114	80	50	27	19.692	19.702 ± 0.0047
